# Interplay of tRNA-Derived Fragments and T Cell Activation in Breast Cancer Patient Survival

**DOI:** 10.3390/cancers12082230

**Published:** 2020-08-10

**Authors:** Nayang Shan, Ningshan Li, Qile Dai, Lin Hou, Xiting Yan, Amei Amei, Lingeng Lu, Zuoheng Wang

**Affiliations:** 1 Center for Statistical Science, Department of Industrial Engineering, Tsinghua University, Beijing 100084, China; sny16@mails.tsinghua.edu.cn (N.S.); houl@tsinghua.edu.cn (L.H.); 2 Department of Biostatistics, Yale School of Public Health, New Haven, CT 06520, USA; ningshan.li@yale.edu (N.L.); qile.dai@yale.edu (Q.D.); xiting.yan@yale.edu (X.Y.); 3 SJTU-Yale Joint Center for Biostatistics and Data Science, Department of Bioinformatics and Biostatistics, School of Life Sciences and Biotechnology, Shanghai Jiao Tong University, Shanghai 200240, China; 4 MOE Key Laboratory of Bioinformatics, School of Life Sciences, Tsinghua University, Beijing 100084, China; 5 Section of Pulmonary, Critical Care and Sleep Medicine, Yale School of Medicine, New Haven, CT 06520, USA; 6 Department of Mathematical Sciences, University of Nevada, Las Vegas, NV 89154, USA; amei.amei@unlv.edu; 7 Department of Chronic Disease Epidemiology, Yale School of Public Health, New Haven, CT 06520, USA

**Keywords:** breast cancer, T cell activation score, tRNA-derived fragments, pathway enrichment analysis, cancer survival

## Abstract

Effector CD8^+^ T cell activation and its cytotoxic function are positively correlated with improved survival in breast cancer. tRNA-derived fragments (tRFs) have recently been found to be involved in gene regulation in cancer progression. However, it is unclear how interactions between expression of tRFs and T cell activation affect breast cancer patient survival. We used Kaplan–Meier survival and multivariate Cox regression models to evaluate the effect of interactions between expression of tRFs and T cell activation on survival in 1081 breast cancer patients. Spearman correlation analysis and weighted gene co-expression network analysis were conducted to identify genes and pathways that were associated with tRFs. tRFdb-5024a, 5P_tRNA-Leu-CAA-4-1, and ts-49 were positively associated with overall survival, while ts-34 and ts-58 were negatively associated with overall survival. Significant interactions were detected between T cell activation and ts-34 and ts-49. In the T cell exhaustion group, patients with a low level of ts-34 or a high level of ts-49 showed improved survival. In contrast, there was no significant difference in the activation group. Breast cancer related pathways were identified for the five tRFs. In conclusion, the identified five tRFs associated with overall survival may serve as therapeutic targets and improve immunotherapy in breast cancer.

## 1. Introduction

Recently, tRNA-derived fragments (tRFs), a novel class of small non-coding RNAs derived from tRNA precursor or mature sequences, have drawn great attention to characterize their biological roles and functional mechanisms in diseases. tRFs play important roles in cancer progression and tumorigenesis [1,2,3,4]. Dysregulation of tRFs have been observed in breast cancer [5,6,7,8]. For example, a novel class of tRFs induced by hypoxic stress was found to bind to the oncogenic RNA-binding protein *YBX*1 and inhibit the stability and expression of multiple oncogenic transcripts by displacing their 3′ UTRs from *YBX*1, which resulted in an antagonized protein activity and suppressing breast cancer progression [5]. Another study found that a number of tRNA-derived small RNAs were abundant in estrogen receptor (ER)-positive breast cancer cell lines, but not in ER negative breast cancer or other tissue cell lines, suggesting that tRFs are involved in cell proliferation of sex hormone-dependent cancers [6]. tRFs were also found to be upregulated in trastuzumab-resistant breast cancer cell lines compared to trastuzumab-sensitive breast cancer and higher levels of tRF expression were correlated with worse progression-free survival in human epidermal growth factor receptor 2 (HER2) positive patients, indicating that tRFs are involved in patient response to trastuzumab-targeted therapy in HER2 positive breast cancer [7]. Several tRFs were identified to be predictive biomarkers for recurrence-free survival in triple-negative breast cancer [8]. Taken together, these findings suggest a functional role of tRFs in breast cancer development and progression.

CD8^+^ T cell exhaustion and enhanced regulatory T cell function have been known to be involved in the progression of human cancer including breast cancer. Once infiltrated T cells are primed and activated by tumor neoantigens to become effector T cells, they will execute their cytotoxic activities to kill tumor cells. Neoantigens are present in tumor cells and are generated from tumor-specific somatic DNA alterations that lead to changes of protein sequences. Neoantigens bind to MHC class I molecules and then are distinguished by CD8^+^ T cells, triggering antitumor immunity. Although tRFs were found to be abundant in immune cells and may play an important role in the immune response [9,10], the underlying mechanisms are not well understood. One possible mechanism is that tRFs regulate gene expression within immune cells; and the other can be that tRFs are recognized by Toll-like receptors to induce the immune responses of Th1 and toxic T lymphocytes [11].

Our previous work reported that the T cell activation score was positively associated with improved survival in breast cancer patients [12,13]. However, it is unclear whether and how interactions between expression of tRFs and T cell activation affect breast cancer patient survival. In this study, we aimed to investigate the effect of tRFs and their interactions with T cell activation status on patient survival in breast cancer, which will advance the understanding of biological mechanisms of tRFs and serve as therapeutic targets in breast cancer.

## 2. Results

### 2.1. Association of tRFs with Patient Survival

The correlations between expression of each tRF and overall survival were evaluated. A full list of the 232 tRFs with their sequences and *p*-value was displayed in Table S1. Among them, fourteen tRFs with a *p*-value < 0.05 were selected to include in the multivariate Cox regression model. After model selection, eight tRFs were retained in the final model, which adjusted for patient’s age at diagnosis, disease stage, histological type, and T cell activation. Among them, expression levels of three tRFs, tRFdb-5024a, 5P_tRNA-Leu-CAA-4-1, and ts-49, were positively associated with overall survival, and two tRFs, ts-34 and ts-58, were negatively associated (Table 1). A higher level of tRFdb-5024a was significantly associated with decreased risk of death. The adjusted HR was 0.52 (95% CI: 0.37–0.74, *p* < 0.001) for high vs. low expression groups. A higher level of 5P_tRNA-Leu-CAA-4-1 was significantly associated with decreased risk of death. The adjusted HR was 0.55 (95% CI: 0.35–0.87, *p* = 0.011) for high vs. low expression groups. A higher level of ts-34 was significantly associated with increased risk of death. The adjusted HR was 1.62 (95% CI: 1.08–2.44, *p* = 0.019) for high vs. low expression groups. A higher level of ts-49 was significantly associated with decreased risk of death. The adjusted HR was 0.40 (95% CI: 0.17–0.93, *p* = 0.032) for high vs. low expression groups. A higher level of ts-58 was significantly associated with increased risk of death. The adjusted HR was 1.56 (95% CI: 1.10–2.20, *p* = 0.013) for high vs. low expression groups. Patients in the T cell activation group had better overall survival compared to those in the exhaustion group. The adjusted HR was 0.48 (95% CI: 0.27–0.83, *p* = 0.009) for activation vs. exhaustion groups.

### 2.2. Interaction between T Cell Activation Status and tRFs in Patient Survival

The interactions between T cell activation status and expression of tRFs were evaluated in the multivariate Cox regression model, with adjustment for patient’s age at diagnosis, disease stage, and histological type. After model selection, two interaction terms were significant: ts-34 by T cell activation interaction (*p* = 0.040) and ts-49 by T cell activation interaction (*p* = 0.008; Table 2). The other three tRFs, tRFdb-5024a, 5P_tRNA-Leu-CAA-4-1, and ts-58, remained significantly correlated with overall survival. A higher level of tRFdb-5024a was associated with decreased risk of death. The adjusted HR was 0.50 (95% CI: 0.36–0.71, *p* < 0.001) for high vs. low expression groups. A higher level of 5P_tRNA-Leu-CAA-4-1 was associated with decreased risk of death. The adjusted HR was 0.58 (95% CI: 0.37–0.92, *p* = 0.021) for high vs. low expression groups. A higher level of ts-58 was associated with increased risk of death. The adjusted HR was 1.51 (95% CI: 1.07–2.12, *p* = 0.018) for high vs. low expression groups.

**Figure 1:**
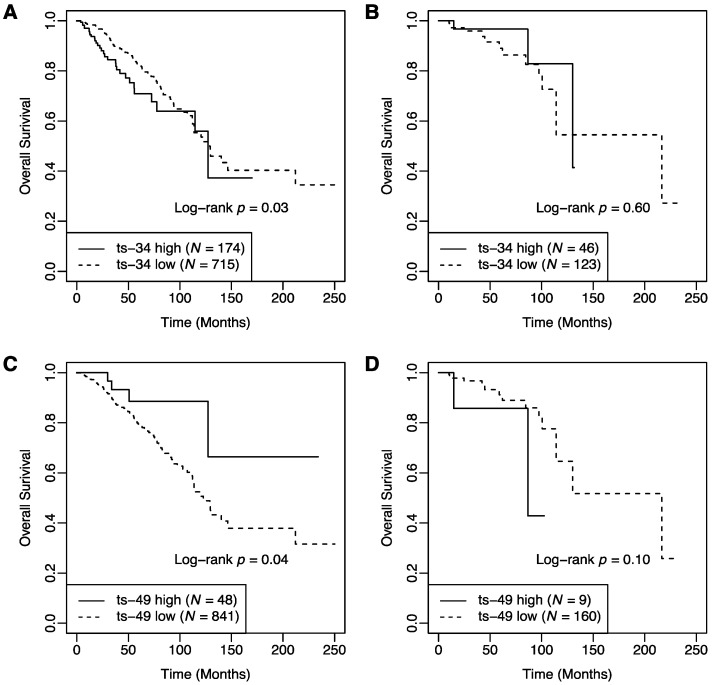
Kaplan–Meier survival curves of breast cancer patients stratified by T cell activation status. (**A**) In the exhaustion group, patients with a low level of ts-34 had better overall survival compared to those with a high level (*p* = 0.03). (**B**) In the activation group, there was no significant difference in overall survival between patients in the high and low levels of ts-34 (*p* = 0.60). (**C**) In the exhaustion group, patients with a high level of ts-49 had better overall survival compared to those with a low level (*p* = 0.04). (**D**) In the activation group, there was no significant difference in overall survival between patients in the high and low levels of ts-49 (*p* = 0.10).

We further investigated the relationship between survival and expression of tRFs stratified by T cell activation status (Table 3). In the exhaustion group, patients with a low level of ts-34 showed better overall survival compared to those with a high level of ts-34 (Figure 1A). The 5-year survival rate was 0.82 (95% CI: 0.78–0.87) in the low expression group (*n* = 715) and 0.71 (95% CI: 0.61–0.82) in the high expression group (*n* = 174). The adjusted HR was 2.13 (95% CI: 1.40–3.23, *p* < 0.001) for high vs. low expression groups. In contrast, among patients in the activation group, there was no significant difference in overall survival between high and low expression groups (Figure 1B). The 5-year survival rate was 0.89 (95% CI: 0.81–0.98) in the low expression group (*n* = 123) and 0.97 (95% CI: 0.90–1.00) in the high expression group (*n* = 46). The adjusted HR was 0.18 (95% CI: 0.03–1.14, *p* = 0.069) for high vs. low expression groups.

Overall survival in high and low groups of ts-49 was different by T cell activation status. In the exhaustion group, improved overall survival was observed in patients with a high level of ts-49 compared to those with a low level of ts-49 (Figure 1C). The 5-year survival rate was 0.79 (95% CI: 0.75–0.84) for the low expression group (*n* = 841) and 0.89 (95% CI: 0.77–1.00) for the high expression group (*n* = 48). The adjusted HR was 0.28 (95% CI: 0.10–0.76, *p* = 0.013) for high vs. low expression groups. In contrast, in the activation group, there was no significant difference in overall survival between high and low expression groups (Figure 1D). The 5-year survival rate was 0.91 (95% CI: 0.85–0.98) for the low expression group (*n* = 160) and 0.86 (95% CI: 0.63–1.00) for the high expression group (*n* = 9). The adjusted HR was 3.91 (95% CI: 0.61–24.95, *p* = 0.150) for high vs. low expression groups.

The associations remained significant between overall survival and tRFdb-5024a, 5P_tRNA-Leu-CAA-4-1, and ts-58 in the exhaustion group. The adjusted HRs were 0.51 (95% CI: 0.35–0.73, *p* < 0.001), 0.54 (95% CI: 0.33–0.88, *p* = 0.014), and 1.58 (95% CI: 1.10–2.26, *p* = 0.013), respectively. In contrast, among patients in the activation group, none of the three tRFs were associated with overall survival.

### 2.3. Correlation between tRFs and Clinical Pathological Variables

The correlations between the expression of the five tRFs and clinical pathological variables, including the ER status, PR status, HER2 status, histological type, and disease stage were assessed (Table S2). Differential expression of tRFdb-5024a was observed among different histological types (*p* = 2.508 × 10^−5^). ts-34 was differentially expressed between ER positive and negative groups (*p* = 1.691 × 10^−6^), between PR positive and negative groups (*p* = 1.424 × 10^−5^), and between HER2 positive and negative groups (*p* = 0.023). ts-58 was differentially expressed between HER2 positive and negative groups (*p* = 1.473 × 10^−4^). There was a borderline significant association between disease stage and ts-49 (*p* = 0.055). 5P_tRNA-Leu-CAA-4-1 was not differentially expressed between any groups of the clinical variables.

### 2.4. Correlation between tRFs and mRNA Transcripts

**Figure 2:**
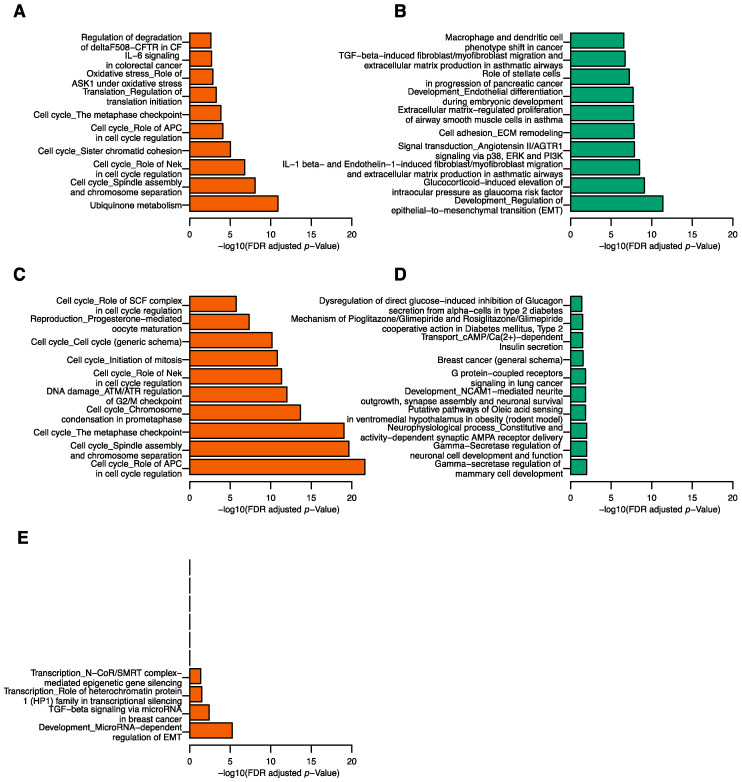
Top gene pathways associated with tRFs. Top 10 pathways from the positively (**A**) and negatively (**B**) correlated genes of tRFdb-5024a, positively (**C**) and negatively (**D**) correlated genes of ts-34, and positively correlated genes of ts-58 (**E**).

The correlations between the five tRFs and 36,674 mRNA transcripts were assessed. We identified 404 positively and 2292 negatively correlated genes with tRFdb-5024a, 16 positively and 2 negatively correlated genes with 5P_tRNA-Leu-CAA-4-1, 310 positively and 230 negatively correlated genes with ts-34, and 280 positively and 1 negatively correlated genes with ts-58. No genes were significantly correlated with ts-49. Pathway enrichment analyses were carried out on positively and negatively correlated genes for the four tRFs separately. For tRFdb-5024a, the positively correlated genes were enriched for 27 pathways and the negatively correlated genes were enriched for 405 pathways (Table S3). Cell cycle related pathways were found in the top 10 pathways enriched in the positively correlated genes (Figure 2A). The top 10 pathways enriched in the negatively correlated genes included epithelial-to-mesenchymal transition (EMT), signal transduction, cell adhesion ECM remodeling, and extracellular matrix-regulated proliferation related pathways (FDR = 4.05 × 10^−12^, 1.40 × 10^−8^, 1.48 × 10^−8^, and 1.77 × 10^−8^, respectively; Figure 2B and for Table S3). For ts-34, the positively correlated genes were enriched for 33 pathways and the negatively correlated genes were enriched for 13 pathways (Table S4). The positively correlated genes were enriched for molecules involved in cell cycle related pathways (Figure 2C), while the negatively correlated genes were involved in mammary cell development and neuronal cell development (Figure 2D). Notably, the most relevant pathway was breast cancer (general schema) pathway in which three genes were negatively correlated with ts-34 (FDR = 2.95 × 10^−2^). For ts-58, four pathways were enriched in the positively correlated genes and no significant pathways were found in the negatively correlated genes (Table S5). The top two pathways were development microRNA-dependent regulation of EMT pathway (FDR = 5.48 × 10^−6^) and TGF-beta signaling via microRNA in the breast cancer pathway (FDR = 3.94 × 10^−3^; Figure 2E). No significant pathways were identified for ts-49 and 5P_tRNA-Leu-CAA-4-1.

### 2.5. Correlation between tRFs and Gene Modules

**Figure 3:**
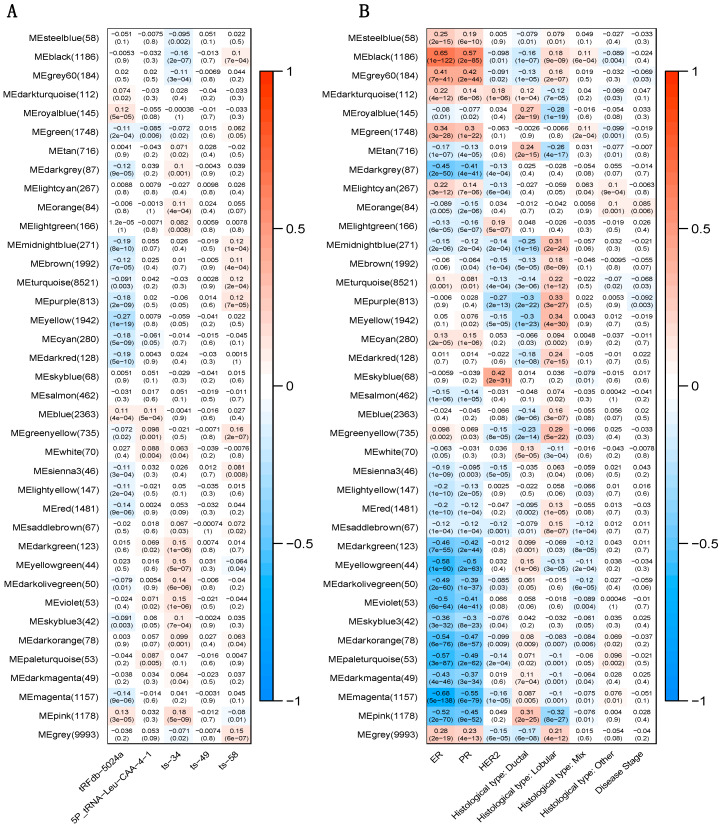
Correlation of gene modules with tRFs and clinical pathological variables. Row represents module eigengenes, column represents tRFs or clinical variables. Each cell displays the corresponding correlation coefficient and *p*-value. (**A**) Heatmap of correlations between module eigengenes and expression of the five tRFs. (**B**) Heatmap of correlations between module eigengenes and clinical variables.

Gene co-expression network was constructed to identify modules of highly correlated genes using weighted gene co-expression network analysis (WGCNA). The identified 38 gene modules and their correlations with expression of the five tRFs were demonstrated in Figure 3A. For tRFdb-5024a, the pink module was the top positively correlated module (r = 0.13, *p* = 3 × 10^−5^) and the yellow module was the top negatively correlated module (r = -0.27, *p* = 1 × 10^−19^). For 5P_tRNA-Leu-CAA-4-1, the top positively correlated module was the blue module (r = 0.11, *p* = 5 × 10^−4^). For ts-34, the pink module was the top positively correlated module (r = 0.18, *p* = 5 × 10^−9^) and the black module was the top negatively correlated module (r = -0.16, *p* = 2 × 10^−7^). For ts-58, the top positively correlated module was the green-yellow module (r = 0.16, *p* = 2 × 10^−7^). Weak correlations were observed for modules that were negatively correlated with 5P_tRNA-Leu-CAA-4-1 and ts-58, which were consistent with the observation that genes were mostly positively correlated with 5P_tRNA-Leu-CAA-4-1 and ts-58. No modules were significantly correlated with ts-49. Figure 3B shows the correlations between gene modules and clinical pathological variables. The pink module was significantly correlated with ER status (r = −0.52, *p* = 2 × 10^−70^), PR status (r = −0.45, *p* = 9 × 10^−52^), ductal subtype (r = 0.31, *p* = 2 × 10^−25^), and lobular subtype (r = −0.32, *p* = 8 × 10^−27^). The yellow module was significantly associated with ductal subtype (r = −0.3, *p* = 1 × 10^−23^) and lobular subtype (r = 0.34, *p* = 4 × 10^−30^). Black module was significantly correlated with ER status (r = 0.65, *p* = 1 × 10^−122^) and PR status (r = 0.57, *p* = 2 × 10^−85^). These results, combined with the significant associations between the tRFdb-5024a and histological type, ts-34 and ER or PR status, suggested potential biological functions of tRFdb-5024a and ts-34 in breast cancer.

**Figure 4:**
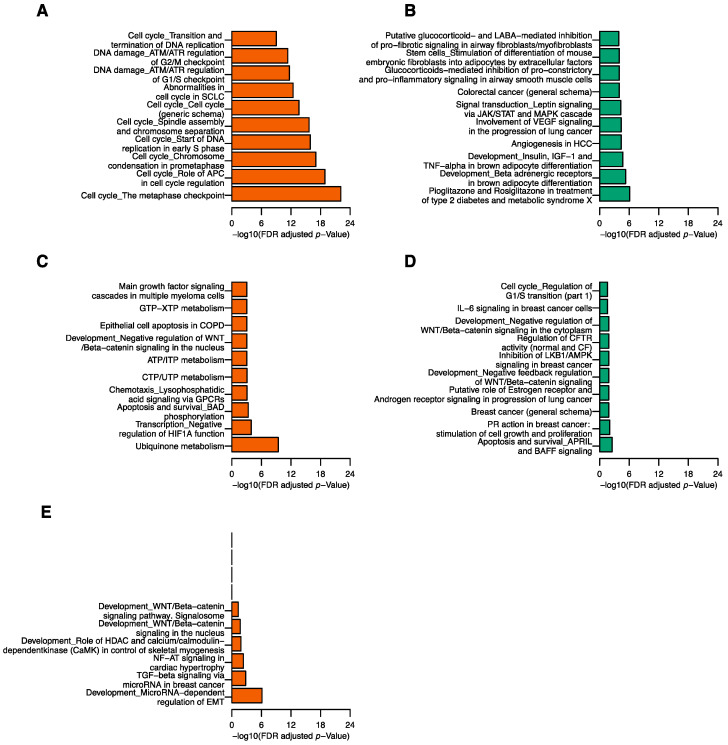
Top pathways of gene modules associated with tRFs. Top 10 pathways enriched in pink (**A**), yellow (**B**), blue(**C**), black (**D**), and green-yellow (**E**) modules.

The pathway enrichment analysis was conducted to evaluate the biological function of top gene modules associated with the five tRFs. There were 80, 197, 159, 31, and 6 pathways enriched in pink, yellow, blue, black, and green-yellow modules, respectively (Table S6). For tRFdb-5024a, genes in the pink module were largely enriched for cell cycle related pathways (Figure 4A), consistent with the pathway results of the positively correlated genes of tRFdb-5024a. The signal transduction pathway enriched in the negatively correlated genes of tRFdb-5024a was also observed in the top 10 pathways of yellow modules (Figure 4B). For 5P_tRNA-Leu-CAA-4-1, blue module (22 genes) was enriched for ubiquinone metabolism pathway (FDR = 3.72 × 10^−10^; Figure 4C and Table S6). Genes such as *NDUFB*1, *NDUFB*2, and *NDUFB*7 were found to have significant prognostic values for breast cancer patients [14]. For ts-34, pathway results of the pink module revealed that many of the top 10 pathways were related to the cell cycle function (Figure 4A), consistent with the pathway results of the positively correlated genes of ts-34. The breast cancer (general schema) pathway was among the top 10 pathways of the black module (Figure 4D), consistent with the pathway results of the negatively correlated genes of ts-34. Interestingly, genes in the black module were enriched for the PR action in breast cancer: stimulation of cell growth and proliferation pathway (FDR = 9.05 × 10^−3^), inhibition of LKB1/AMPK signaling in breast cancer pathway (FDR = 1.37 × 10^−2^), and IL-6 signaling in breast cancer cells pathway (FRD = 2.48 × 10^−2^) (Figure 4D and Table S6). For ts-58, the top two significant pathways of green-yellow module (Figure 4E), development MicroRNA-dependent regulation of EMT and TGF-beta signaling via microRNA in breast cancer, agreed well with the pathway results of the positively correlated genes of ts-58.

## 3. Discussion

tRFs have been found to be dysregulated in several human cancers, such as prostate, colon, lung, and breast cancer [8,15,16,17,18]. However, understanding of the biological mechanisms of tRFs in cancer is still in its infancy. In this study, we explored the effect of tRFs and their interactions with the T cell activation score on patient survival in breast cancer. We also identified genes and modules that were associated with tRFs. Pathway enrichment results provided valuable insights into the functions of tRFs.

To our knowledge, this is the first attempt to investigate how interactions between expression of tRFs and T cell activation status affect breast cancer patient survival using a large cohort available in TCGA. We integrated multiple data types, including mRNA, tRFs, and clinical information. Our results suggest that tRFdb-5024a, 5P_tRNA-Leu-CAA-4-1, and ts-49 were positively associated with patient survival, while ts-34 and ts-58 were negatively associated. ts-34 and ts-49 showed significant interactions with T cell activation status. In the T cell exhaustion group, better overall survival was observed in patients with a low level of ts-34 and a high level of ts-49. However, there was no significant difference in overall survival between high and low expression groups of ts-34 and ts-49 in the T cell activation group. These findings suggest that tRFs affect patient survival differently depending on the T cell activation status. We also observed that tRFdb-5024a was significantly associated with the histological type; ts-34 was significantly associated with ER, PR, and HER2 status; and ts-58 was significantly associated with HER2 status. These results added to the evidence of biological functions of tRFdb-5024a, ts-34, and ts-58 in breast cancer.

**Figure 5:**
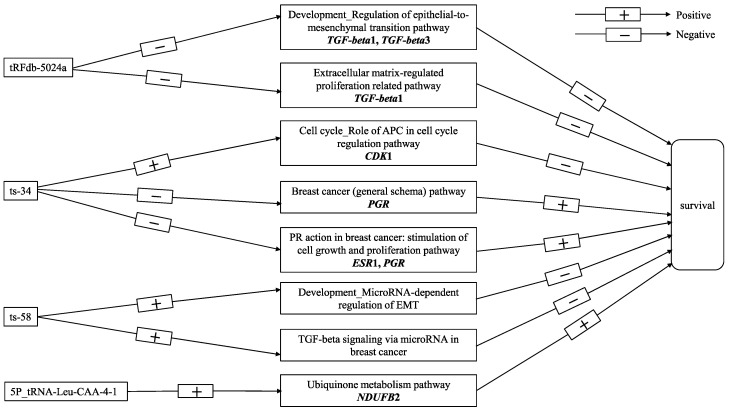
A schematic pathway diagram of the potential molecular mechanisms of vital-associated tRFs in breast cancer.

Spearman correlation analysis demonstrated that tRFdb-5024a mainly downregulates gene expression, whereas 5P_tRNA-Leu-CAA-4-1, ts-34, and ts-58 upregulate gene expression. Gene modules associated with the breast cancer-related tRFs appeared to be involved in various biological processes in human cancer, including cell cycle, EMT, extracellular matrix-regulated proliferation, signal transduction, neuronal cell development, and mammary cell development. Our study showed that negatively correlated genes with tRFdb-5024a (e.g., *TGF-beta*1 and *TGF-beta*3) were enriched for development regulation of the EMT pathway and extracellular matrix-regulated proliferation related pathway. EMT is a hallmark of human cancer with the acquisition of cancer stem cell-like traits that promote cancer progression and metastasis [19]. Extracellular matrix is a major structural component of the tumor microenvironment and serves as a niche for cancer stem cells. It influences the recruitment of immune cells, impairs proliferation and activation of T cells, and induces EMT [20]. Previous studies showed that extracellular matrix-dependent disruption of cell adhesion could lead to increased cell motility and circulating tumor cells, consequently resulting in elevated relapse and mortality risk in breast cancer [21,22]. The cell cycle role of APC in the cell cycle regulation pathway was enriched in both positively correlated genes of tRFdb-5024a and ts-34, suggesting that tRFdb-5024a and ts-34 may target different genes in the cell cycle pathway. Cell cycle-related genes include oncogenes and tumor suppressor genes. For example, Anaphase-Promoting Complex or Cyclosome (APC/C) is an E3 ubiquitin ligase and functions as a tumor suppressor by degrading CDKs [23], whereas high expression of *CDK*1 was previously reported to be correlated with poor survival [24]. Thus, it warrants further investigation of which distinct target gene(s) in the cell cycle pathway is targeted by tRFdb-5024a and ts-34. Interestingly, we found that ts-34 downregulated genes were enriched for breast cancer (general schema) pathway, where absent expression of *PGR* was correlated with poor survival [25]. Similarly, the WGCNA analysis showed that the ts-34 negatively correlated black module was enriched for the PR action in breast cancer: stimulation of cell growth and proliferation pathway. Absent expression of *ESR*1 and/or *PGR* showed resistance to hormonal therapy and higher risk of mortality [25,26]. The top two significant pathways, development microRNA-dependent regulation of EMT and TGF-beta signaling via microRNA in breast cancer, were enriched in both the positively correlated genes and green-yellow module of ts-58. This is consistent with the reports in the previous studies that high expression of *TGF-beta* and miR-21 was positively associated with poor prognosis in breast cancer [27,28]. The underlying mechanisms may involve the upregulation of development microRNA-dependent regulation of the EMT pathway and TGF-beta signaling via microRNA in breast cancer, which increases metastasis by promoting EMT [29]. In addition, the WGCNA results demonstrated new insights into potential functions of 5P_tRNA-Leu-CAA-4-1. The top significant pathway, ubiquinone metabolism pathway, was enriched in the positively correlated blue module of 5P_tRNA-Leu-CAA-4-1, where high expression of *NDUFB*2 had a better prognostic value in breast cancer patients [14]. Figure 5 illustrates a schematic pathway diagram that presents the potential molecular mechanisms of vital-associated tRFs in breast cancer in this study. The biological functions of ts-49 remains to be explored in the future.

There are several caveats and limitations in this study. First, molecular subtype information is available on a small subset of patients, preventing us from incorporating it into the analysis. Whether and how the effect of tRFs and their interactions with the T cell activation status on patient survival varying with different molecular subtypes remains to be investigated in the future. Second, chemotherapy information is unavailable in this study so that we cannot include chemotherapy as a covariate in the multivariate Cox regression models. Thus, interpretation and generalization of the results need to be cautious. However, as the standard care for breast cancer is surgical resection followed by adjuvant chemotherapy for all patients except for those who were too sick to tolerate chemotherapy, leading to a relatively small proportion of patients without chemotherapy in clinic practice, our results might be robust given that the sample size is relatively large in this study.

## 4. Materials and Methods

### 4.1. Study subjects and Data Sources

Included in this study were 1081 female patients with primary breast cancer. Their clinical data were obtained from The Cancer Genome Atlas (TCGA) breast invasive carcinoma study [30]. The normalized RNA sequencing data were downloaded from the TCGA-BRCA project in Genomic Data Commons (GDC) data portal [31], which provided expression levels of 60,483 mRNA transcripts. T cell activation scores were computed based on 13 genes related to T cell activation status (*NKG*7, *CCL*4, *CST*7, *PRF*1, *GZMA*, *GZMB*, *IFNG*, *CCL*3, *PD*-1, *TIGIT*, *LAG*3, *TIM*3, and *CTLA*4), as described previously [12]. To obtain expression of tRFs, small RNA raw sequencing reads were processed for quality control and then realigned to the human reference genome (hg19) and the human genomic tRNA database [32,33]. We used normalized count matrix consisting of 232 distinct tRFs from the tRFexplorer database in our analysis [34,35].

One thousand and fifty-eight patients had clinical, mRNA gene expression, and tRFs data, and were retained for further analysis. The average age at diagnosis was 58.4 (range: 26–90) years old. Among the 1048 patients with disease stage information, most were diagnosed with breast cancer at an early stage including 178 (17.0%) at stage I and 600 (57.3%) at stage II. The other 270 patients (25.7%) were diagnosed at an advanced stage (III or IV). There were 1056 patients with histological type information: 71.5% (*n* = 755) ductal carcinoma, 18.8% (*n* = 198) lobular, 4.9 % (*n* = 52) mix, and 4.8% (*n* = 51) other. Among the 1009 women with a known ER status, 77.0% (*n* = 777) were positive and 23.0% (*n* = 232) were negative. Among the 1006 patients with a known progesterone receptor (PR) status, 67.0% (*n* = 674) were positive and 33.0% (*n* = 332) were negative. There are 700 patients with HER2 information: 22.3% (*n* = 156) were positive and 77.7% (*n* = 544) were negative. The average length of follow-up in the 1058 patients was 40.8 (range: 0–282.7) months and 147 patients died during the follow-up period.

### 4.2. Statistical Analyses

Multivariate Cox proportional hazards models were used to evaluate the relationship between the expression of tRFs and overall survival, adjusting for covariates including the patient’s age at diagnosis, disease stage, histological type, and T cell activation status. Both the expression of tRFs and the T cell activation score were treated as categorical variables in the analysis. For each tRF, patients were classified into two groups, high and low, with the cutoff value to be the median of the expression level. T cell activation status, activation or exhaustion, was defined as previously described [12]. We first selected a small set of tRFs with a *p*-value < 0.05 by the Cox regression model with a single tRF adjusting for covariates. Then the multivariate Cox model with backward elimination was conducted to select significant tRFs and obtain adjusted hazards ratios (HRs) with 95% confidence intervals (95% CIs). The interaction between T cell activation status and expression of tRFs were evaluated by including it in the Cox regression model as an interaction term. Backward elimination using Akaike information criterion was performed for model selection. We also assessed effects of tRFs on overall survival in each subgroup stratified by the T cell activation status using Kaplan–Meier survival curves. Proportional hazards assumption was examined in the analysis. For each clinical pathological variable, Wilcoxon test and Kruskal–Wallis test were used to evaluate differential expression of tRFs between patients from different pathological categories. In all statistical analyses, *p*-value less than 0.05 was considered to be significant. All analyses were performed in R [36].

### 4.3. Functional Pathway Analysis

To understand the biological functions of tRFs in breast cancer progression, we performed functional pathway analysis using the MetaCore software [37]. Spearman correlation analysis was carried out to assess correlations between significant tRFs identified in the Cox regression models and mRNA expression levels. Positively and negatively correlated genes with a Bonferroni corrected *p*-value < 0.05 were included in a pathway analysis separately. We also performed WGCNA to construct gene correlation networks and identify modules of highly correlated genes [38]. In the WGCNA analysis, we first excluded genes with no variation (standard deviation of fragments per kilobase million (FPKM) = 0) or with low expression (FPKM < 0.05) in 90% of the samples. Then, we clustered patients based on their gene expression profiles and removed sample outliers. Lastly, we applied WGCNA with the default setting to obtain gene modules and their correlation with significant tRFs and clinical pathological variables. Genes in top modules with the highest positive or negative correlation coefficients with each tRF and *p*-value < 0.001 were selected for the pathway analysis. Fisher’s exact test was used to determine whether a gene module is enriched for a functional pathway. Pathways with a false discovery rate (FDR) < 0.05 were considered to be significant. We reported pathways that are significant and more than one gene overlapped with the gene module [39].

## 5. Conclusions

In this study, we integrated multiple data types to elucidate the effect of tRFs and their interactions with T cell activation score on patient survival in breast cancer and to uncover the biological relevance of tRFs with statistical significance. We identified five tRFs that were significantly associated with patient survival in breast cancer, especially in the T cell exhaustion group, suggesting that these five tRFs are potential therapeutic targets to improve patient survival, and may have implication in improving immunotherapy in breast cancer. Moreover, our results provided novel knowledge in biological functions of the tRFs in breast cancer.

## Figures and Tables

**Table 1:** Relationships between overall survival and tRNA-derived fragments (tRFs).

Variables		Death	
	HR	95% CI	*p*-Value
T Cell Activation			
Exhaustion	1.00		
Activation	0.48	0.27–0.83	0.009
tRFdb-5024a			
Low	1.00		
High	0.52	0.37–0.74	*p* < 0.001
5P_tRNA-Leu-CAA-4-1			
Low	1.00		
High	0.55	0.35–0.87	0.011
ts-34			
Low	1.00		
High	1.62	1.08–2.44	0.019
ts-49			
Low	1.00		
High	0.40	0.17–0.93	0.032
ts-58			
Low	1.00		
High	1.56	1.10–2.20	0.013
tRFdb-1040			
Low	1.00		
High	1.49	0.93–2.38	0.096
5P_tRNA-Ala-AGC-8-2			
Low	1.00		
High	1.49	0.99–2.27	0.059
ts-13			
Low	1.00		
High	1.38	0.94–2.03	0.103
Age (per 5 years)	1.21	1.13–1.29	*p* < 0.001
Disease Stage			
Stage I	1.00		
Stage II	2.38	1.31–4.31	0.004
Stage III or IV	7.03	3.84–12.85	*p* < 0.001
Histological type			
Ductal	1.00		
Lobular	0.55	0.34–0.87	0.011
Mix	0.59	0.28–1.24	0.161
Other	2.38	1.24–4.57	0.009

**Table 2:** Interactions between T cell activation status and tRFs in the whole sample.

Variables		Death	
	HR	95% CI	*p*-Value
T Cell Activation			
Exhaustion	1.00		
Activation	0.60	0.32–1.12	0.110
tRFdb-5024a			
Low	1.00		
High	0.50	0.36–0.71	*p* < 0.001
5P_tRNA-Leu-CAA-4-1			
Low	1.00		
High	0.58	0.37–0.92	0.021
ts-34			
Low	1.00		
High	2.12	1.40–3.22	*p* < 0.001
ts-49			
Low	1.00		
High	0.27	0.10–0.74	0.011
ts-58			
Low	1.00		
High	1.51	1.07–2.12	0.018
T cell Activation × ts-34	0.22	0.05–0.94	0.040
T cell Activation × ts-49	13.49	2.00–91.02	0.008
Age (per 5 years)	1.20	1.12–1.28	*p* < 0.001
Disease Stage			
Stage I	1.00		
Stage II	2.18	1.21–3.94	0.010
Stage III or IV	6.35	3.50–11.52	*p* < 0.001
Histological type			
Ductal	1.00		
Lobular	0.53	0.33–0.83	0.006
Mix	0.56	0.26–1.19	0.130
Other	2.60	1.34–5.02	0.005

**Table 3:** Relationships between overall survival and tRFs stratified by T cell activation status.

Stratification Variable	Variables	Death		
		HR	95% CI	*p*-Value
**T cell Exhaustion group**	tRFdb-5024a			
	Low	1.00		
	High	0.51	0.35–0.73	*p* < 0.001
	5P_tRNA-Leu-CAA-4-1			
	Low	1.00		
	High	0.54	0.33–0.88	0.014
	ts-34			
	Low	1.00		
	High	2.13	1.40–3.23	*p* < 0.001
	ts-49			
	Low	1.00		
	High	0.28	0.10–0.76	0.013
	ts-58			
	Low	1.00		
	High	1.58	1.10–2.26	0.013
	Age (per 5 years)	1.21	1.13–1.30	*p* < 0.001
	Disease Stage			
	Stage I	1.00		
	Stage II	2.60	1.35–4.99	0.004
	Stage III or IV	7.18	3.72–13.86	*p* < 0.001
	Histological type			
	Ductal	1.00		
	Lobular	0.49	0.30–0.80	0.004
	Mix	0.51	0.23–1.12	0.094
	Other	2.16	0.98–4.76	0.056
**T cell Activation group**	tRFdb-5024a			
	Low	1.00		
	High	0.57	0.15–2.06	0.388
	5P_tRNA-Leu-CAA-4-1			
	Low	1.00		
	High	0.55	0.12–2.49	0.442
	ts-34			
	Low	1.00		
	High	0.18	0.03-1.14	0.069
	ts-49			
	Low	1.00		
	High	3.91	0.61–24.95	0.150
	ts-58			
	Low	1.00		
	High	0.50	0.14–1.81	0.291
	Age (per 5 years)	1.16	0.94–1.43	0.157
	Disease Stage			
	Stage I	1.00		
	Stage II	0.53	0.11–2.71	0.449
	Stage III or IV	4.37	0.74–25.68	0.103
	Histological type			
	Ductal	1.00		
	Lobular	0.99	0.19–5.32	0.999
	Mix	3.65	0.33–40.56	0.292
	Other	6.21	1.39–27.71	0.017

## Supplementary Materials

The following are available online at https://www.mdpi.com/2072-6694/12/8/2230/s1, Table S1: A full list of tRFs with their sequences and *p*-value, Table S2: Differential expression of tRFs between patients from different pathological categories, Table S3: Significant pathways enriched in positively and negatively correlated genes of tRFdb-5024a, Table S4: Significant pathways enriched in positively and negatively correlated genes of ts-34, Table S5: Significant pathways enriched in positively correlated genes of ts-58, Table S6: Significant pathways enriched in pink, yellow, blue, black, and green-yellow modules.
